# The Course and Effects of Syphllis When Untreated by Any Remedies

**Published:** 1868-01

**Authors:** 


### The Course and Effects of Syphilis when Untreated by any Remedies.

We have reason to believe that the papers which have appeared from time to time in this journal, have done something towards advancing the knowledge of syphilitic diseases in this country; and we take an opportunity of stating afresh, in a concise way, what we have mainly endeavored to inculcate. While mercurialists have ranged themselves in two opposing factions, and have been energetically arguing their respective claims, and advancing their own line of facts, it would almost seem as if one important matter had been well nigh lost sight of altogether. Syphilis is a disease depending upon the evolution and development of a specific blood poison, and like all the other diseases with which it is in these respects allied, it is liable to great variety in the severity and character of its morbid manifestations. Nobody is likely to deny this with regard to small-pox and scarlatina, for example. In these disorders such differences are everywhere recognized, and we accordingly speak of any given case as simple, benign or malignant, according to the severity or otherwise of its symptoms. Those who have enjoyed the largest field for the observation of syphilitic disorders, and who have had the best opportunities for watching the natural evolution of that disease, cannot avoid perceiving that we want less of statement and more of results impartially drawn from a series of comparative observations. A great deal of hasty generalization would then be swept away, and we should not have men confounding the natural products of a severe type of syphilis, with those artificially induced processes resulting from mercury. It can not be too strongly urged that syphilis tends to run a pretty regular and definite course, and that great differences exist in the severity of the manifestations in different cases, irrespective of drugs—specific or others—that the milder forms of the disorder frequently recover under all plans of treatment; that some cases—where there is no such disturbing element present as mercury to accuse as the cause—are extremely severe, and a few even die from pure syphilis or of the asthenia induced by that disease; and lastly, that it seems to be practically pretty well settled that mercury, on the whole, is, nevertheless, the most reliable agent we possess for its treatment. While we quite agree with those who think that mercury is capable of exerting a baneful influence in some constitutions, and in certain forms, stages, and variations of this disease, and while we would extend the most ample field for liberty of action, and regard skepticism in all matters not yet proved, as the first step towards getting them proved, we do not consider extravagant statements, advanced by advocates on one side or the other, calculated to lead to any good, and least of all to the good of science. It was fortunate for one individual who, disbelieving in such a thing as syphilis at all, and for a great number who refer all the worst forms of it to the drugs employed, that some of the members of the late Admiralty Commission, we learn, had the opportunity of seeing a soldier patient in whom the constitutional effects of syphilis were so formidable that he died of that disease within six months of contracting the primary lesion; and in that case, at any rate, no mercury whatever had been exhibited. We gather some important facts from Dr. Paynter's report on the French troops serving in Algeria, bearing on this subject of the effects observed in the natural evolution of the disease. The paper is to be found in the sanitary section of the new Army Medical Blue-book. Dr. Paynter says that among the native Arab populatiou syphilis is the only really prevalent disease, both in the towns and in the country districts. As a general rule these people never seek any advice or treatment. and one meets with most dreadful objects suffering from the malady in its various forms. Extensive disease of the bones, where no kind of treatment had been at any time adopted, is frequently met with amidst these people. The appearance of some of the sufferers, even to those accustomed to witness disease, is described as most revolting. One French army surgeon had seen a native woman, the bones of whose face had been completely ulcerated away by the disease now under consideration. She had never received any treatment whatever, either for the primary or other stages of the affection. Where these native people first contracted this disease, or how it was introduced among them, it is difficult to form any idea; but it is quite evident that it exists to a most lamentable extent, and may, with other causes too numerous and varied to define, eventually and at no distant period, exterminate the subdued tribes from the face of this most beautiful country.—*London Medical Times and Gazette, November* 30, 1867.

